# Antiviral Activity of Berbamine Against Influenza A Virus Infection

**DOI:** 10.3390/ijms26062819

**Published:** 2025-03-20

**Authors:** Won-Kyung Cho, Hee-Jeong Choi, Jin Yeul Ma

**Affiliations:** Korean Medicine (KM) Application Center, Korea Institute of Oriental Medicine, 70 Chemdanro, Dong-gu, Daegu 41062, Republic of Korea; chj1901@kiom.re.kr

**Keywords:** berbamine (BBM), anti-influenza A virus, hemagglutinin (HA), virucidal effect

## Abstract

Berbamine (BBM) is a bibenzyl isoquinoline present in the traditional Chinese herbal medicine *Berberis amurensisis* Rupr. The present study demonstrates that BBM exerts strong antiviral efficacy against influenza A virus (IAV) infection. We examined the anti-IAV effect of BBM using green fluorescent protein (GFP)-expressing influenza A and H1N1 IAV. The fluorescence microscopy, fluorescence-activated cell sorting analysis, and plaque assay showed that BBM significantly hinders IAV infection. The immunofluorescence analysis confirmed the anti-influenza activity of BBM. From the time-of-addition and hemagglutination inhibition results, it is elucidated that the antiviral effect of BBM is closely related to its inhibitory effect against viral binding and entry at an early infection stage. Our findings imply that BBM has the potential to be developed as a potent antiviral drug against influenza viral infection.

## 1. Introduction

Influenza viruses belong to the Orthomyxoviridae family and have a segmented RNA genome, causing respiratory and systemic symptoms such as sore throat, fever, cough, headache, and muscular pain, severely affecting the high-risk group up to death [1]. Influenza A viruses (IAVs) are the main cause of seasonal flu epidemics annually [2]. Frequent antigenic shifts and antigenic point mutations during RNA replication generate new IAV variants with different types of hemagglutinin (HA) and/or neuraminidase (NA) [3]. It is impossible to predict new influenza strains in advance and develop a perfect vaccine. M2 protein inhibitors such as rimantadine and amantadine have been used as antiviral agents clinically. However, they do not protect against infection from influenza B viruses and are resistant to influenza A viruses [4]. Currently, neuraminidase inhibitors, such as zanamivir, oseltamivir, and peramivir, and RNA polymerase, inhibitors such as baloxavir, are being used clinically; however, side effects and drug resistance to these inhibitors are being reported [5,6].

Berbamine (BBM) is a bibenzyl isoquinoline present in the traditional Chinese herbal medicine *Berberis amurensisis* Rupr. [7]. BBM has been reported to possess various pharmacological effects such as anti-cancer [8,9,10,11], anti-inflammatory [12], anti-hypercholesterolemic [13], anti-osteoporosis [14], and anti-DSS-induced colitis [15]. Recently, several researchers discovered that BBM has antiviral efficacy against the African swine fever virus (ASFV) [16], Ebola virus [17], SARS-Cov-2 [18], Equine herpesvirus type 1 [19], Porcine epidemic diarrhea virus [20], bovine viral diarrhea virus [21], and Japanese encephalitis virus (JEV) [22]. In this study, we demonstrate for the first time that BBM exhibits potent anti-influenza virus infection by blocking viral binding to the cells through the inhibition of hemagglutination in the early stages of IAV infection.

## 2. Results

### 2.1. Cytotoxicity of Berbamine (BBM)

**Figure 1:**
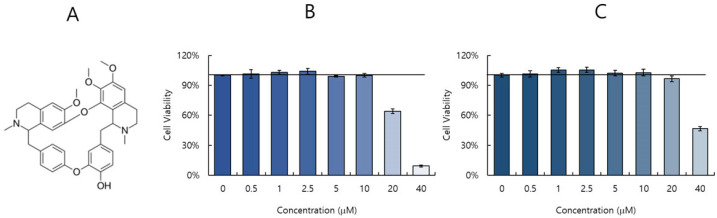
The structure (**A**) and toxicity of BBM in RAW 264.7 (**B**) and A549 (**C**) cells. The cells were treated with BBM at the indicated concentrations for 24 h at 37 °C. A CCK-8 assay was used to examine the cytotoxicity of BBM in the cells. The data represent the mean ± SD based on three replicates in three different experiments.

The cytotoxicity of BBM on the cells was evaluated by a CCK-8 assay. When the cell viability was checked at 24 h post-treatment with BBM at the indicated concentrations, BBM did not show cytotoxicity up to 10 µM (Figure 1B,C).

### 2.2. Berbamine Dose-Dependently Inhibits Influenza Virus Infection

**Figure 2:**
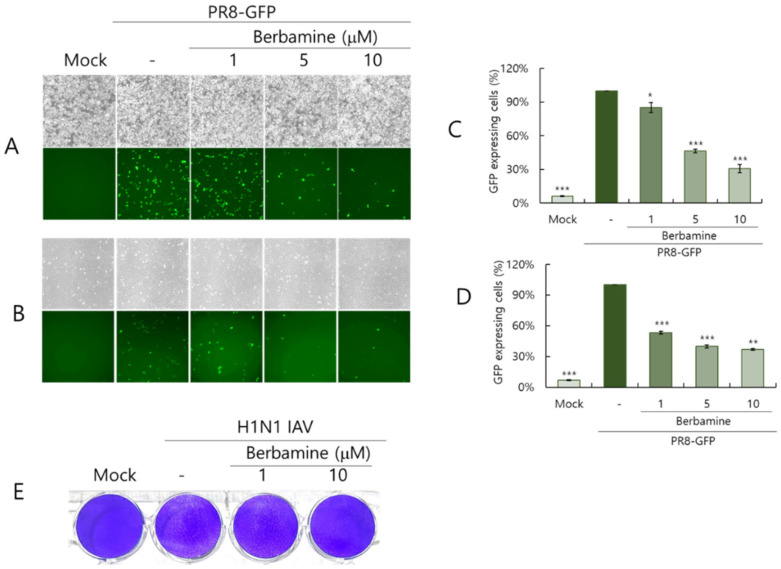
Antiviral effect of BBM against influenza A viral infection. (**A**–**D**) PR8-GFP IAV (10 MOI) and BBM (0, 1, 5, or 10 μM) were mixed and preincubated for 1 h at 4 °C. The mixtures were added to RAW 264.7 (**A**,**C**) or A549 (**B**,**D**) cells for 2 h at 37 °C. After washing, the cells were further incubated for 24 h at 37 °C. The brightfield and fluorescence images were captured under the fluorescence microscope (200× magnification). The cells were fixed and analyzed by FACS to compare the relative GFP-expressing cells among each group. The data represent the mean ± SD based on three independent experiments. (**E**) H1N1 IAV and BBM (1 or 10 μM) were incubated at 4 °C for 1 h and added to MDCK cells at 37 °C for 1 h. The cells were washed with PBS, overlaid with 1.5% agarose-containing DMEM, and further incubated for 72 h. The cells were fixed and stained with 1% crystal violet. The data represent the mean ± SD based on three independent experiments. Statistical significance was assessed via an unpaired Student *t*-test. *** *p* < 0.001, ** *p* < 0.005, * *p* < 0.05 compared with PR8-GFP IAV-infected group.

We used the GFP-expressing influenza A/PR8/34 (PR8-GFP IAV) virus to examine the anti-influenza viral effect of BBM. First, when we tested whether BBM has an inhibitory effect against the influenza virus infection using pretreatment, co-treatment, and post-treatment approaches in RAW 264.7 cells, we found that BBM showed strong antiviral efficacy in the co-treatment trial (Supplementary Figure S1). To confirm the effect of BBM on IAV infection in more detail, BBM at 1, 5, or 10 uM and PR8-GFP IAV were added to RAW 264.7 and A549 cells for 24 h. Figure 2A,B present the RAW 264.7 and A549 cells infected by PR8-GFP IAV expressed GFP, whereas the cells in the presence of BBM dose-dependently significantly decreased GFP expression. To confirm the antiviral effect of BBM on IAV infection, we conducted FACS analysis on the cells infected by PR8-GFP IAV in the absence or presence of BBM. The numbers of GFP-expressing cells in each group were compared with the virus-infected control group (Figure 2C,D). Consistent with the results in Figure 2A,B, BBM potently reduced GFP expression by PR8-GFP IAV infection. Next, we explored whether BBM could inhibit wild-type H1N1 IAV infection using a plaque assay in MDCK cells. When we infected MDCK cells with H1N1 IAV without or with BBM (1 or 10 μM), we found that BBM at 10 μM significantly reduced plaque formation by H1N1 IAV, as shown in Figure 2E.

### 2.3. Immunofluorescence Results Confirmed the Anti-Influenza Viral Effect of Berbamine

**Figure 3:**
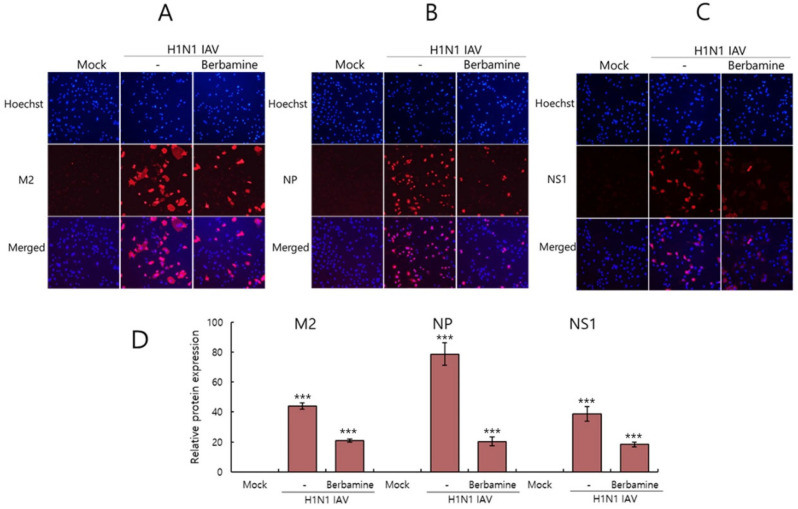
Effect of BBM on viral protein expression in the IAV-infected cells. A549 cells were co-treated with H1N1 IAV at an MOI of 10 and 5 µM BBM for 24 h. The cells were fixed for 10 min and immuno-stained with antibodies against M2 (**A**), NP (**B**), or NS1 (**C**) protein (Red) for 1 h. The nuclei were stained with Hoechst 33342 (blue color) for 10 min. The co-localization of viral proteins and nuclei was visualized under a fluorescence microscope. The red viral protein expressions were quantified and represented the mean ± SD based on triplicate experiments (**D**). Statistical significance was assessed via an unpaired Student *t*-test. *** *p* < 0.001 compared with the non-infected group.

Next, we checked the IAV protein expression in the presence of BBM to confirm the inhibitory effect of BBM on IAV infection. We performed an immunofluorescence analysis on A549 cells infected with H1N1 IAV in the presence or absence of BBM. The infected cells were fixed with paraformaldehyde and stained with Alexa 594-tagged antibodies specifically for IAV M2, NS1, and NP proteins. Hoechst 33342 was used to detect nuclei. Significantly lowered levels of M2, NP, and NS1 proteins were detected in the presence of BBM (Figure 3). These results confirm that BBM inhibits IAV infection, thereby reducing viral protein expression, consistent with Figure 1.

### 2.4. Berbamine Exerts a Virucidal Effect and the Blockage of Virus Binding and Entry on the Cells

**Figure 4:**
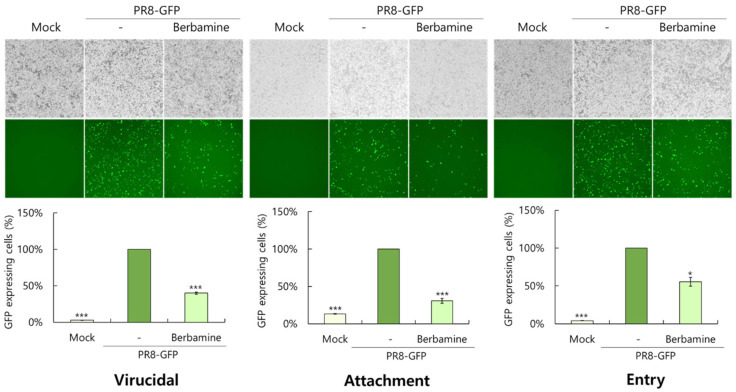
Effect of BBM on IAV infection at the early stages. The time-of-addition experiment was conducted under different incubation times and temperature conditions to infect cells with the PR8-GFP IAV and BBM, as described in the Materials and Methods section in detail. The cell images were captured using fluorescence microscopy with 200× magnification. The levels of GFP-expressing cells were analyzed using FACS. The data represent the mean ± SD value based on three independent experiments. Statistical significance was assessed via an unpaired Student’s *t*-test. *** *p* < 0.001, * *p* < 0.05 compared with PR8-GFP IAV-infected group.

Given that BBM markedly inhibits IAV infection, we used a time-of-addition assay with a GFP-expressing virus and BBM to determine which IAV infection stages during infection are affected by BBM. Because influenza viruses can only bind to the cell surface and cannot enter the cells at 4 °C, but IAV could penetrate the cells at 37 °C, different incubation times and temperatures (4 °C or 37 °C) were used to co-treat IAV and BBM at each stage. To check the virucidal effect of BBM, PR8-GFP IAV and BBM were coincubated for 30 min at 4 °C, and the mixtures were administered to the cells for 30 min at 37 °C. After washing with PBS to remove the virus and BBM, the cells were further incubated for 24 h at 37 °C. To investigate the effect of BBM on virus attachment to the cells, PR8-GFP IAV and BBM were co-administered to the cells for 30 min at 4 °C. The cells were washed with PBS and incubated for 24 h at 37 °C. To examine viral entry into the cells, the cells were infected by PR8-GFP IAV for 30 min at 4 °C and washed with PBS. After being treated with BBM for 30 min at 37 °C and washed with PBS, the cells were further incubated for 24 h at 37 °C. The inhibitory effect of BBM on IAV infection at each stage was determined through GFP expression compared to the PR8-GFP virus infection group. First, when we tested whether BBM could directly kill viruses before binding to the cells, we found that BBM had a strong virus-eradicating effect, as presented in Figure 4, in the left panel. Next, when we checked the effect of BBM on viral binding and penetration, BBM significantly blocked IAV infection at both stages. These results suggest that BBM has a potent inhibitory effect against influenza virus by preventing viral infection at an early stage.

### 2.5. Berbamine Dose-Dependently Suppresses Hemagglutination

**Figure 5:**
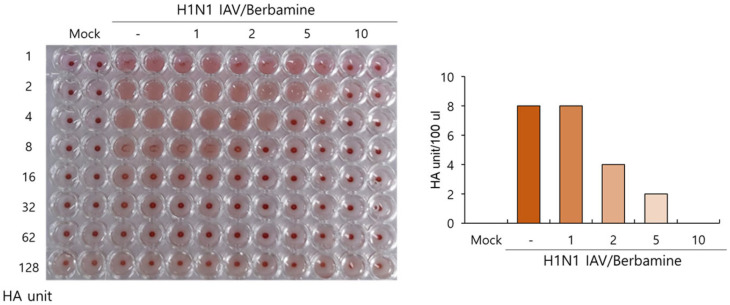
Effect of BBM on hemagglutination caused by IAV infection. BBM (0, 1, 2, 5, or 10 µM) was mixed with H1N1 IAV for 1 h at 4 °C, and each mixture was added to the cells for 24 h. The serially diluted supernatants were mixed with 1% RBCs. The data show a representative image of three independent experiments.

Since BBM showed a significant inhibitory effect on influenza viral attachment and entry into the cells (Figure 4), we investigated the effect of BBM on hemagglutination by IAV HA proteins. HA is an indispensable protein for IAV binding and entry to the cells and is known to induce the hemagglutination of RBC. As shown in Figure 5, in the absence of BBM, the H1N1 virus caused hemagglutination, while BBM significantly inhibited virus-induced hemagglutination from 2 µM to 10 µM, dose-dependently. The H1N1 virus-infected control expressed 8 HA units. HA units in the presence of 2 µM or 5 µM of BBM were 2-fold or 4-fold lower than the virus control, respectively. Notably, 10 µM of BBM completely prevented the hemagglutination. These results suggest that BBM potently represses the HA protein, impairing IAV binding and entry into the cells.

### 2.6. Berbamine Did Not Affect Neuraminidase Activity

**Figure 6:**
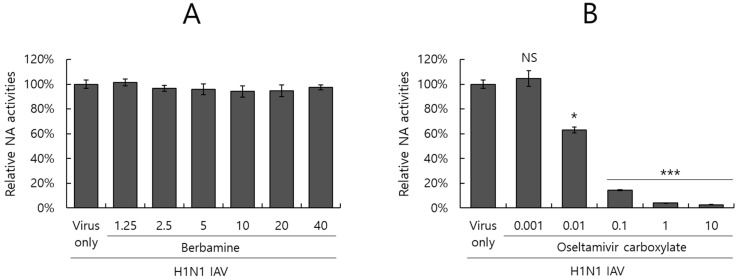
Effect of BBM on IAV neuraminidase activity. According to the manufacturer’s instructions, BBM (**A**) or oseltamivir carboxylate (**B**) was mixed with H1N1 IAV and subjected to a neuraminidase inhibition assay. The NA activities were detected with an excitation wavelength of 365 nm and an emission wavelength of 445 nm. The data represent the mean ± SD based on three independent experiments. Statistical significance was assessed via an unpaired Student’s *t*-test. *** *p* < 0.001, * *p* < 0.05 compared with H1N1 virus-infected group. NS; no significance.

Given that the neuraminidase of the influenza virus is known to facilitate progeny release from the infected cell and another target for antiviral drugs, we examined whether BBM could affect the NA activity of IAV. According to the NA-Fluor Influenza Neuraminidase assay protocol, the inhibitory effect on NA activity was assessed using serially diluted BBM or oseltamivir carboxylate, as a positive control, and H1N1 IAV. While oseltamivir carboxylate showed a dose-dependent inhibitory effect on NA of H1N1 IAV (Figure 6B), BBM did not affect neuraminidase activity (Figure 6A). These results suggest that BBM did not prevent the release of viral progeny from the infected cells at the later stages of IAV infection.

## 3. Discussion

Influenza viruses are seasonal and mutate, posing a significant threat to public health. Antivirals such as neuraminidase and RNA polymerase inhibitors are currently in use, but several side effects have been discovered, prompting the development of new treatments. In this study, we investigated whether BBM has inhibitory activity against influenza viruses and its potential as an antiviral agent. The antiviral efficacy of BBM against influenza viruses was examined using GFP expression caused by PR8-GFP influenza viruses and plaque formation caused by PR8-H1N1 influenza viruses. The fluorescence microscopy and FACS analyses with or without BBM presented that BBM significantly dose-dependently inhibited IAV infection in both the RAW 264.7 and A549 cells (Figure 2A–D). The plaque inhibition assay confirmed the antiviral effect of BBM against H1N1 IAV infection (Figure 2E). Based on the results showing a strong inhibitory effect of BBM upon influenza virus infection, we conducted a time-of-addition assay to determine whether BBM prevents viral binding and entry to the cells in the early infection stage. Notably, BBM significantly blocked viral binding and entry to the cells and exerted potent virus-eradication efficacy (Figure 4). BBM reduced virus binding by more than 2-fold compared to the virus-infected control. BBM inhibited viral penetration into cells by more than 70% compared to the virus-infected group. Further, BBM showed a potent virucidal effect before binding and entering the cells. Influenza virus infection is initiated by the binding of the virus’s hemagglutinin to the sialic acid-linked glycoprotein receptors on cells in the early infection stages of IAV [23]. Many studies have been conducted to inhibit viral infection by blocking the binding of sialic acid to HA or by inhibiting HA. Several research results suggest that chemicals with triterpene [24] and pentacyclic triterpene structures [25] could inhibit viral binding and HA activity, thereby preventing IAV infection. Sialic acid inhibitors targeting HA through computer modeling have also been studied to discover anti-influenza viral agents [26]. Several compounds in natural products have been reported as anti-influenza virus agents by modulating HA activity and inhibiting viral binding to cells. Neoechinulin B has been reported to have an inhibitory effect on WSN/33 H1N1 IAV in both co-treatment and post-treatment trials and to inhibit HA and NA activity [27]. Aureonitol was known to have an inhibitory effect on H3N2 IAV and only HA without inhibiting NA [28]. Amentoflavone [29] and Isoquercitrin [30], discovered in our previous studies, showed inhibitory effects on PR8/34 H1N1 and H3N2 IAV and inhibited both HA and NA activities. Ginsenoside rk1 has been reported to have inhibitory effects on H1N1, H3N2, and H5N2 IAV [31]. The hemagglutination inhibition assay showed that BBM dose-dependently repressed hemagglutination and 10 µM BBM completely prevented hemagglutination (Figure 5). These results suggest that BBM may prevent IAV from binding to the cell membrane by inhibiting the HA of IAV. Although BBM did not affect the neuraminidase activity of IAV, it showed a strong anti-influenza viral effect by inhibiting the early stages of viral infection, including HA interference and virus-eradicating efficacy. Our results suggest that BBM may be used as a potent influenza virus inhibitor by combining it with NA inhibitors such as oseltamivir and zanamivir.

## 4. Materials and Methods

### 4.1. Materials, Cell Culture, and Viruses

Berbamine dihydrochloride (PubChem CID: 275182) purchased from Sigma-Aldrich (St. Louis, MO, USA) was dissolved in DMSO. A549 cells (human lung adenocarcinoma cell) and RAW 264.7 cells (Mouse Leukemic Monocyte Macrophage cell line; ATCC TIB-71) were maintained in RPMI medium (Hyclone, Logan, UT, USA) in the presence of fetal bovine serum (10%) and Penicillin and Streptomycin (100 U/mL) at 37 °C with 5% CO_2_. Green fluorescent protein (GFP)-expressing influenza A/PR8/34 (PR8-GFP) and A/PR8/34 (H1N1) viruses were kindly provided by Dr. Jong-Soo Lee (Chungnam National University, Daejeon, Republic of Korea). HBPV-VR-32 (H3N2) influenza virus was obtained from the Korea Bank for Pathogenic Viruses (KBPV). The viruses were propagated using a 10-day-old chicken embryo. All virus-related experiments were conducted under Biosafety Level 2.

### 4.2. Cell Viability Assay

RAW 264.7 cells (1 × 10^5^ cells/well) and A549 cells (5 × 10^4^ cells/well) were seeded in 96 wells and incubated for 24 h. Cells were treated with BBM at final concentrations ranging from 1 to 40 µM for 24 h. The cells were added with 10 μL of CCK-8 reagent (Dojindo, Rockville, MD, USA) for 2 h, and then the absorbance at 450 nm was measured using an ELISA microplate reader (Promega, Madison, WI, USA).

### 4.3. Anti-Influenza Viral Assay

BBM (1, 5, or 10 µM) and influenza A/PR8-GFP virus (PR8-GFP IAV), at an MOI of 10, were mixed for 1 h at 4 °C. The mixtures were added to A549 or RAW 264.7 cells for 2 h at 37 °C. After washing with PBS, the cells were further incubated for 24 h. Fluorescent microscopy or fluorescence-activated cell sorting (FACS) was used to detect the GFP-expressing cells. To analyze the GFP expression of cells, the cells infected with PR8-GFP IAV in the presence or absence of BBM were harvested and fixed with 4% paraformaldehyde. The cells were resuspended in PBS and analyzed by a CytoPLEX flow cell counter (Beckman Coulter Inc., Pasadena, CA, USA) to examine GFP expression. To perform the plaque reduction assay, MDCK cells at a density of 5 × 10^5^ cells per well were seeded in 12-well cell culture plates. H1N1 IAV was mixed with BBM (0, 1, or 10 µM) at 4 °C for 1 h. The mixtures were added to MDCK cells and incubated for 2 h at 37 °C. After washing with PBS, the cells were overlaid with a 1.5% agar-containing DMEM medium and incubated for 72 h. The cells were fixed with 4% paraformaldehyde for 10 min. After removing the agar overlay, the cells were stained with 1% crystal violet.

### 4.4. Time-of-Addition Assay

We examined the effect of BBM on IAV attachment, entry, and virucidal before binding to the cells using the PR8-GFP expression system and different incubation conditions. To investigate the effect of BBM on attachment, PR8-GFP IAV at an MOI of 10 and 5 µM of BBM were added to RAW 264.7 cells for 30 min at 4 °C. After washing with PBS, the cells were incubated for 24 h at 37 °C. To examine the effect on entry, the cells were incubated with PR8-GFP IAV for 30 min at 4 °C, followed by the addition of BBM for 30 min at 37 °C. After washing, the cells were incubated for 24 h at 37 °C. To check the virucidal effect, BBM and PR8-GFP IAV were mixed for 30 min at 4 °C. The mixtures were co-administered to the cells for 30 min at 37 °C. After washing, the cells were further incubated for 24 h at 37 °C. The microscopic images were visualized under fluorescence microscopy with 200× magnification. To compare the GFP-expressing cells among groups, the cells were fixed with 4% paraformaldehyde for 10 min, resuspended in PBS, and FACS-analyzed.

### 4.5. Immunofluorescence

A549 cells were added with a mixture of H1N1 IAV at an MOI of 10 and 5 µM of BBM, which were preincubated for 1 h at 4 °C. The cells were fixed with 4% paraformaldehyde for 10 min at 24 h post-infection. After blocking with 1% BSA-PBS, the cells were incubated with antibodies (GeneTex, Irvine, CA, USA) against influenza viral proteins for 12 h at 4 °C. After washing with PBS containing 0.05% Tween 20, the cells were incubated with Alexa Fluor 594-conjugated secondary antibody (Invitrogen, Waltham, MA, USA) for 1 h at 37 °C in the dark. Hoechst 33342 (Invitrogen, Waltham, MA, USA) was added for 5 min to stain the nuclei. The red viral proteins and blue nuclei images were colocalized using fluorescent microscopy.

### 4.6. Hemagglutination Inhibition Assay

BBM at the indicated concentrations was mixed with H1N1 IAV for 1 h at 4 °C. The mixtures were added to RAW 264.7 cells for 24 h at 37 °C. The supernatant of cells was serially diluted by two-fold and added to a 96-well U-bottom plate. An equal volume of 1% chicken Red Blood Cells (RBCs) (Innovative Research, Inc., Southfield, MI, USA) in PBS was added to each well and incubated for 1 h. RBCs in the absence of the virus exhibited aggregation, and RBCs in the virus-infected well were hemolyzed. HA titers were determined as HA units/100 µL in comparison with the virus control.

### 4.7. Neuraminidase Inhibition Assay

The NA-Fluor Influenza Neuraminidase Assay Kit (Life Technologies, Carlsbad, CA, USA) was used to determine the effect of BBM on neuraminidase activity. BBM, serially diluted by 2-fold from 40 μM to 1.25 μM, was added to 96-well black plates and mixed with H1N1 IAV. Oseltamivir carboxylate (Aobious Inc., Gloucester, MA, USA), a specific NA inhibitor, was diluted from 10 μM to 0.001 μM and used as a positive control. After incubating for 30 min, NA-Fluor Substrate was then added to the mixture for 1 h at 37 °C. The reaction was terminated using an NA-Fluor stop solution. The neuraminidase activity was measured by a fluorescence spectrometer (Promega, Madison, WI, USA) at an excitation wavelength of 365 nm and an emission wavelength of 445 nm.

## 5. Conclusions

BBM protects cells from IAV infection. It inhibits IAV binding and entry into the cells via the modulation of IAV hemagglutinin. BBM shows a significant virus-eradication effect before entering the cells. Our results show that BBM can be used as an antiviral agent to suppress influenza virus infection in the early stages.

## Data Availability

The data are contained in the article.

## Supplementary Materials

The following supporting information can be downloaded at https://www.mdpi.com/article/10.3390/ijms26062819/s1.
