# Supplementary material for: Antiviral Activity of Berbamine Against Influenza A Virus Infection
Source: Int J Mol Sci. 2025 Mar 20;26(6):2819. doi: 10.3390/ijms26062819 (PMC11942913; doi:10.3390/ijms26062819)
Supplement: Supplementary file 1 [file ijms-26-02819-s001.zip › ijms-3523818-supplementary.pdf]

Supplementary Materials Data

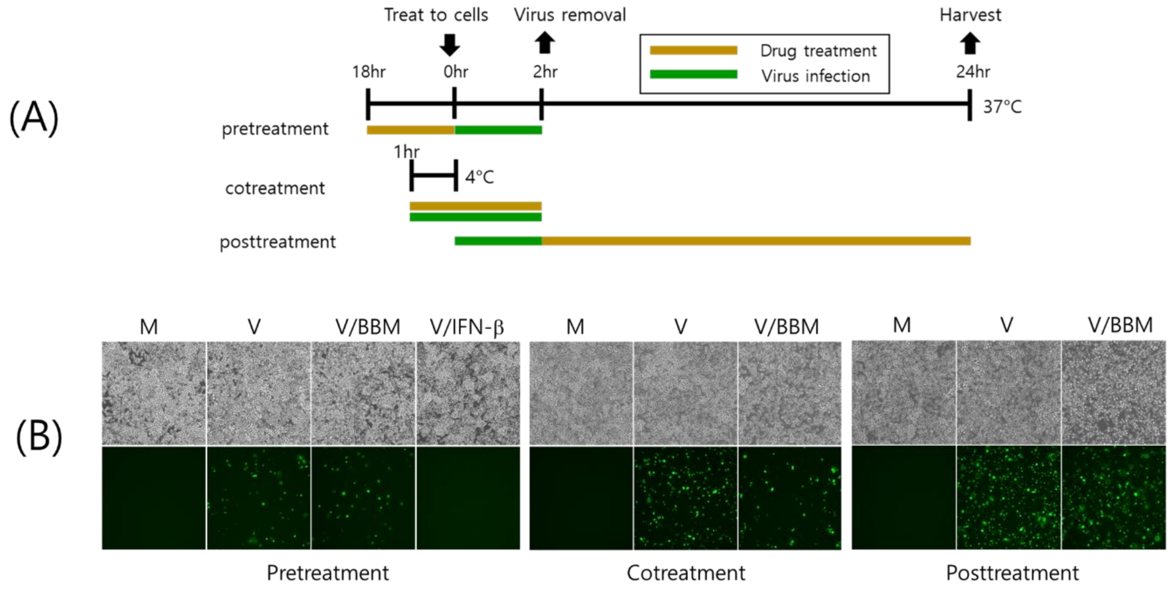

Supplementary Figure S1. (**A**) The scheme of pretreatment, cotreatment, and posttreatment trials of BBM and PR8-GFP IAV in RAW 264.7 cells. (**B**) PR8-GFP IAV and BBM were administered to the RAW 264.7 cells, as indicated in Figure 1A. The GFP expression of infected cells was observed using fluorescence microscopy. M: untreated control; V: PR8-GFP IAV-infected; V/BBM: PR8-GFP IAV- and BBM-treated; V/IFN-, PR8-GFP IAV-, and IFN-β (as used for positive antiviral control)-treated.
